# Locomotion characteristics of a wheeled vibration-driven robot with an enhanced pantograph-type suspension

**DOI:** 10.3389/frobt.2023.1239137

**Published:** 2023-08-11

**Authors:** Vitaliy Korendiy, Oleksandr Kachur

**Affiliations:** Department of Technical Mechanics and Engineering Graphics, Institute of Mechanical Engineering and Transport, Lviv Polytechnic National University, Lviv, Ukraine

**Keywords:** inertial vibration exciter, unbalanced rotor, kinematic characteristics, locomotion conditions, oscillatory system, numerical modeling, computer simulation

## Abstract

**Introduction:** The paper considers the improved design of the wheeled vibration-driven robot equipped with an inertial exciter (unbalanced rotor) and enhanced pantograph-type suspension. The primary purpose and objectives of the study are focused on mathematical modeling, computer simulation, and experimental testing of locomotion conditions of the novel robot prototype. The primary scientific novelty of the present research consists in substantiating the possibilities of implementing the enhanced pantograph-type suspension in order to improve the robot’s kinematic characteristics, particularly the average translational speed.

**Methods:** The simplified dynamic diagram of the robot’s oscillatory system is developed, and the mathematical model describing its locomotion conditions is derived using the Euler-Lagrange differential equations. The numerical modeling is carried out in the Mathematica software with the help of the Runge-Kutta methods. Computer simulation of the robot motion is performed in the SolidWorks Motion software using the variable step integration method (Gear’s method). The experimental investigations of the robot prototype operating conditions are conducted at the Vibroengineering Laboratory of Lviv Polytechnic National University using the WitMotion accelerometers and software. The experimental data is processed in the MathCad software.

**Results and discussion:** The obtained results show the time dependencies of the robot body’s basic kinematic parameters (accelerations, velocities, displacements) under different operating conditions, particularly the angular frequencies of the unbalanced rotor. The numerical modeling, computer simulation, and experimental investigations present almost similar results: the smallest horizontal speed of about 1 mm/s is observed at the supplied voltage of 3.47 V when the forced frequency is equal to 500 rpm; the largest locomotion speed is approximately 40 mm/s at the supplied voltage of 10 V and forced frequency of 1,500 rpm. The paper may be interesting for designers and researchers of similar vibration-driven robotic systems based on wheeled chassis, and the results may be used while implementing the experimental and industrial prototypes of vibration-driven robots for various purposes, particularly, for inspecting and cleaning the pipelines. Further investigation on the subject of the paper should be focused on analyzing the relations between the power consumption, average translational speed, and working efficiency of the considerer robot under various operating conditions.

## 1 Introduction

Among a great variety of mobile robots, vibration-driven locomotion systems are ofparticular attention due to their design specificity and characteristic operating regimes. Numerous scientific papers are devoted to so-called bristle-type or brush-type robots. Paper (Cicconofri and DeSimone, 2015) presents a thorough analysis of the motion conditions of a vibration-driven robot with rigid bristles, and an unbalanced rotor-type exciter is carried out. A comprehensive study on the dynamics of a bristle-type system moving along a horizontal shacking plate is presented in Cicconofri et al. (2016). A millimeter-scale vibration-driven robot with a bristle-type suspension and a piezoelectric actuator is considered in Kim et al. (2020a). The novel design of the bristle-type platform made of magnetic material is experimentally tested in Kim et al. (2020b). Paper (Choi et al., 2020) is dedicated to studying the dynamic behavior and solving the parameters optimization problem of an in-pipe robot with elastic bristles. The research on the influence of bristles inclination angle on the robot’s motion conditions is conducted in Robles-Cuenca et al. (2020). An interesting design of a brush-type locomotion system equipped with a DC motor driving the unbalanced rotor is considered in Tallapragada and Gandra (2021). Another direction of development of the bristle-type robots is focused on combining several independent robotic units (modules) into a single locomotion system. An example of such a design is studied in (Diao et al., 2022; Diao et al., 2021) from the viewpoint of optimizing its control system to improve locomotion performance. The possibilities of implementing bristle-type multi-module systems in the processes of inspecting and cleaning the pipelines are substantiated in Gmiterko et al. (2017). Similarly to the bristle-type robots, the stick-slip locomotion principle can be provided in the crawling systems with an elastic body set into the oscillatory motion due to the periodic disturbance generated by the enhanced dielectric-elastomer actuator (Calabrese et al., 2019). The same actuator is implemented in an enhanced design and experimental prototype of the vibro-impact crawling robot equipped with tilted bristles, whose locomotion conditions are thoroughly studied in (Wu et al., 2022).

Another interesting type of vibration-driven robot is based on a capsule-type locomotion system. The latest investigations on these systems are presented in thousands of publications. Among the most common areas of implementing such robotic systems, diagnosis and endoscopy of internal organs of a human body (Hanscom and Cave, 2022; Jiang et al., 2022) and inspecting the pipelines (Verma et al., 2021; Jang et al., 2022) are of the most investigated ones. A comprehensive review of electromagnetically-driven vibratory robotic systems is presented in (Chen et al., 2022). In most cases, such robots are designed on the basis of double-mass vibro-impact systems able to slide along tubes, pipes, or intestines (Liao et al., 2022). Computer simulation and experimental studies of the capsule-type robot’s dynamic behavior under the action of the external magnetic field are carried out in (Zhang et al., 2022). Paper (Yan et al., 2022) is dedicated to solving the optimization problem aimed at improving the locomotion conditions of the self-propelling robotic system. In (Yin et al., 2022), the authors expanded their investigations by the bifurcation analysis of the previously studied robot during its sliding along the small intestine. A novel actuator with three permanent magnets and one solenoidal coil is proposed and experimentally tested in (Wu and Lu, 2022). An interesting design of a capsule-type robot with an active electromechanical transmission driving the leg-type locomotion mechanisms is developed and implemented as a laboratory prototype in (Sun et al., 2022). Another set of investigations dedicated to the vibration-driven locomotion systems was initiated in (Duong et al., 2018), where the authors substantiated the general idea of the robot structure based on Duffing oscillator. Papers (Nguyen et al., 2020; NguyenDu and La, 2020) supplement previous research by applying the vibro-impact working regimes, which were theoretically and experimentally studied. In (Nguyen et al., 2021; La et al., 2021), the authors comprehensively investigated the influence of various friction types on the capsule-type system dynamic behavior under different operating conditions (with and without impact modes).

Unlike the bristle-type, crawling-type, and capsule-type robots, the vast majority of vibration-driven locomotion systems are based on the wheeled chassis. The authors of the present research started working on the development of vibration-driven locomotion systems at the end of 2021 when the initial idea of implementing the twin (doubled) crank-slider excitation mechanism in a semidefinite vibratory system was stated (Korendiy et al., 2021). Based on this idea, the mathematical model of the wheeled vibration-driven locomotion system was derived and numerically solved in (Korendiy et al., 2022a), whilst the initial design of the wheeled robot was developed and simulated in (Korendiy et al., 2022b). The experimental investigations on the motion conditions of the considered robot were performed in (Korendiy et al., 2022c), and the influence of the impact-gap value on the robot’s translational speed was analyzed in (Korendiy et al., 2022d). Similar investigations were held by numerous researchers worldwide (Rusu and Tatar, 2022; Tagliavini et al., 2022). For example, in (Xie et al., 2021), the authors considered a wheeled in-pipe robot equipped with an enhanced actuating mechanism based on synchronized cam-linkage and sliding-rotating units. Another interesting design of a wheeled self-propelled robot containing two movable frames able to slide relative to one another is thoroughly studied in (Feng et al., 2020). Paper (Jia et al., 2021) considers the enhanced design of the wheeled robot equipped with the piezoelectric actuator that allows for improving its performance.

In distinction to various crank-type, electromagnetic, and piezoelectric actuators of the wheeled vibration-driven robots, they are commonly equipped with inertial exciters based on unbalanced rotors. A theoretical background on the dynamics of the locomotion systems driven by inertial vibration exciters is presented in (Bolotnik et al., 2006). An interesting design of the wheeled robot with a kinematically synchronized doubled unbalanced exciter was considered in (Loukanov, 2015). Further experimental investigations on this robot’s dynamic behavior were conducted in (Loukanov and Stoyanov, 2015). Papers (Loukanov et al., 2016a; Loukanov et al., 2016b) supplement the previous investigations by the numerical modeling of the robot motion conditions, whilst in (Loukanov, 2016), the authors developed a novel approach of multi-criteria synthesis of the wheeled vibration-driven robot parameters. Along with the wheeled locomotion systems, unbalanced rotors are widely used for actuating capsule-type and in-pipe robots. An interesting research on the optimal control of a capsule system with an unbalanced pendulum is presented in (Zarychta et al., 2022). Similar investigations dedicated to the dynamic behavior of a capsule-type robot with a pendulum-type exciter are carried out in (Liu et al., 2018a). The paper (Liu et al., 2018b) supplements previous results by the optimization algorithms aimed at providing the robot adaptive tracking control. In (Liu et al., 2019), the authors expanded their studies with different friction models. Another set of investigations dedicated to a mobile robotic platform driven by two independent unbalanced rotors was initiated in (Vartholomeos and Papadopoulos, 2008), where the basic motion characteristics were studied. A few years later, the authors focused their attention on the control system of the considered vibration-driven robot (Vartholomeos et al., 2013) and analyzed two different approaches to providing the desired motion trajectory (Vlachos et al., 2015). Considering the robots intended for inspecting the pipelines, the enhanced driving mechanism based on two unbalanced rotors synchronized by a non-circular gear transmission is studied in (Liu et al., 2022). A wide range of investigations is dedicated to developing self-propelled compacting machines based on vibration-driven robotic systems, eliminating the additional forces needed to be applied by the operator to push the corresponding equipment (Korendiy et al., 2022e; Korendiy and Kachur, 2023). The inertial type vibration exciters designed in the form of unbalanced rotors can be effectively combined with additional flywheels improving the locomotion performance of vibration-driven robots and simplifying their control systems (Dosaev et al., 2021; Dosaev, 2022).

The present research continues the authors’ previous investigations, which are published in (Korendiy et al., 2022f; Korendiy et al., 2022g; Korendiy et al., 2023) and focused on theoretical and experimental studying of the dynamic behavior of the wheeled vibration-driven robots equipped with inertial exciters (unbalanced rotors) and rod-type suspensions. In this paper, the enhanced pantograph-type mechanism is proposed to be implemented as the robot’s suspension. The primary purpose and objectives of the present study are focused on mathematical modeling, computer simulation, and experimental testing of locomotion conditions of the novel robot prototype in order to propose practical recommendations on choosing the appropriate excitation parameters and providing the prescribed kinematic characteristics. The major scientific novelty of the present research consists in substantiating the possibilities of implementing the enhanced pantograph-type suspension in order to improve the wheeled vibration-driven robot’s operating efficiency, particularly the average translational speed. This paper is divided into eight basic sections. The first one presents the general design of the wheeled vibration-driven robot with inertial vibration exciter and pantograph-type suspension. The second section is dedicated to constructing the simplified dynamic diagram of the robot’s oscillatory system and deriving differential equations that model its locomotion conditions. The description of the experimental technique and equipment is presented in the third section. The fourth one contains the results of numerical modeling of the system vibrations carried out in the Mathematica software. In the fifth section, the computer simulation results obtained in the SolidWorks software are presented. Experimental data and its processing results are introduced in the sixth section. The seventh one presents the general discussion and comparative analysis of the obtained results. The conclusions on the carried out investigations, their practical value, and prospects of further research on the subject of the present paper are stated in the eighth section.

## 2 Research methodology

### 2.1 General design of the wheeled vibration-driven robot with inertial vibration exciter and pantograph-type suspension

**FIGURE 1:**
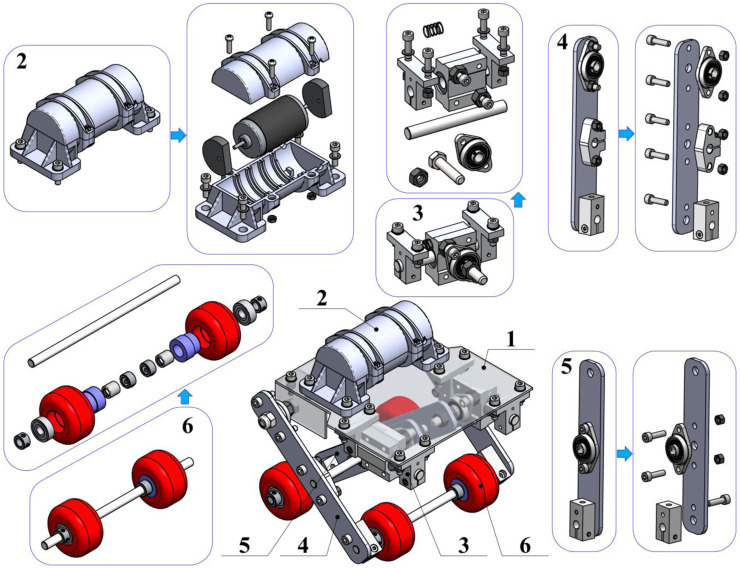
General design of the wheeled vibration-driven robot with pantograph-type suspension: **(1)**—main plate (robot’s body); **(2)**—vibration exciter; **(3)**—spring-slider system; **(4)**, **(5)**—rods (levers) of the pantograph-type suspension; **(6)**—wheeled axles.

As has already been mentioned above, this paper considers the improved design of the wheeled robot driven by an unbalanced vibration exciter (Figure 1). The main design components (assemblies) of the robot are the following: the main body (mounting plate) 1; inertial vibration exciter (unbalanced rotor) 2; spring-slider system 3; rods (levers) 4 and 5 of the pantograph-type suspension; wheeled axles 6. The plate 1 is used for installing the exciter 2 and some elements of the robot’s suspension (e.g., guiding rods, connecting axles, etc.). The vibration exciter 2 consists of a DC motor fixed inside a molded plastic body. The unbalanced masses are fixed on both ends of the motor’s shaft. The spring-slider system 3 contains four clamps fixed on the main plate 1. The clamps hold (restrain) the axles along which the linear (guide) bearings move. The sliders’ motion is restricted by the coil cylindrical springs.

The outer levers (rods) 4 of the pantograph-type suspension are hinged to the main plate with the help of the ball-bearing units. In the middle sections and the lower ends of the outer levers 4, the clamps are installed to fix the corresponding axles, which connect the outer levers 4 with the inner levers (rods) 5 and the wheeled axles 6, respectively. The inner rods 5 are hinged to the guide-bearing units of the spring-slider systems with the help of the ball-bearing units. The same units are installed in the middle sections of the rods 5 to join them with the intermediate axle connecting the outer and the inner levers. The clamps are installed on the lower ends of the inner levers to fix the corresponding wheeled axles 6. The latter consists of two wheels installed on the corresponding axle with the help of the ball bearing and the overrunning (free-wheel) clutch. In order to restrict the possible sliding of the wheels along the axle, additional clamps are fixed on both sides of the wheels.

The robot’s motion can be described as follows. The rotation of the DC motor’s shaft generates centrifugal forces acting upon the unbalanced masses. Under steady-state motion conditions, these forces are characterized by a periodically changed direction, while their magnitude remains almost constant. The excited oscillations of the robot’s body take place in the vertical plane. The horizontal components of the centrifugal forces provide the robot locomotion along the supporting surface when their direction coincides with the permissible (allowable) direction of the robot motion defined by the overrunning clutches. If the direction of the centrifugal forces is opposite to the permissible motion direction, the robot decelerates or stops. On the other hand, the vertical components of the centrifugal forces excite vertical oscillations of the robot’s body and actuate the pantograph-type suspension. When the body moves down, the rear wheels are blocked by the overrunning clutches, and the front wheels move forward. If the robot’s body starts moving up, the front wheels remain blocked while the rear wheels move forward. In such a case, all the components of the centrifugal forces are used to generate the robot locomotion. The main purpose of this study is to substantiate the possibilities and expediency of implementing the proposed pantograph-type suspension for increasing the robot’s average translational speed.

### 2.2 Kinematic diagram and mathematical model of the robot’s oscillatory system

**FIGURE 2:**
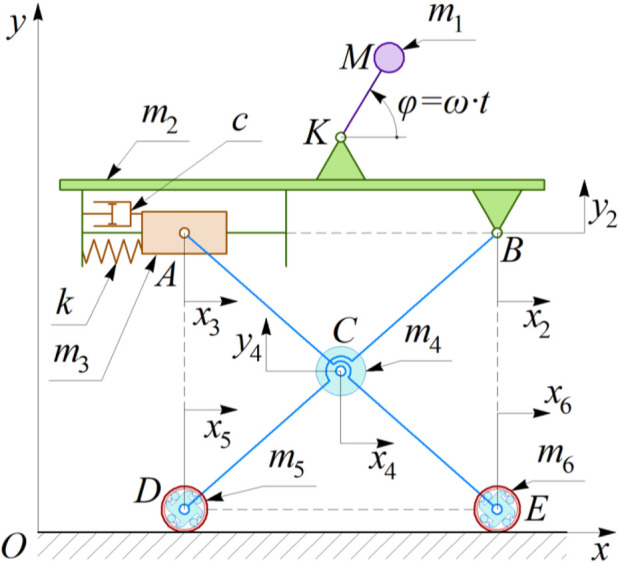
Simplified kinematic diagram of the robot’s oscillatory system.

The simplified kinematic diagram of the robot’s oscillatory system is presented in Figure 2. The system consists of a rigid body of the mass *m*_2_, on the top plane of which the unbalanced mass *m*_1_ rotates around the hinge *K* at a radius $r=l_{KM}$ . The coordinate *φ* describes the instantaneous angular position of the crank *KM* with respect to the horizontal axis. Considering the constant angular speed *ω* of the DC motor’s shaft under the steady-state operating conditions, the angle *φ* can be calculated as follows: φ=ω∙t , where *t* denotes time. The robot’s body (*m*_2_) is suspended from the horizontal supporting surface by the pantograph-type mechanism *ABCDE*. The lever (rod) *BD* is hinged to the robot’s body at *B*, while the overrunning clutch *D* joins it to the rear wheel. The lever (rod) *AE* is hinged to the slider *A*, while its lower end is joined to the overrunning clutch *E* installed in the front wheel. The overrunning clutches allow the wheels to rotate in a clockwise direction while the robot’s leftward motion is restricted. The masses of the rear and front wheeled axles are *m*_5_ and *m*_6_, respectively. The slider *A* of the mass *m*_3_ reciprocates relative to the robot’s body under the action of the spring *k* and damper *c*. The middle sections of the levers *AE* and *BD* are hinged at the point *C*. Let us consider that the mass *m*_4_ located at the point *C* denotes the total mass of all the levers and the axle connecting them.

To model the robot’s locomotion conditions, the inertial coordinate system *xOy* is adopted. The coordinates *x*_2_ and *y*_2_ describe the robot’s body’s horizontal and vertical position relative to the inertial coordinate system *xOy*. The displacement of the slider *A* with respect to the coordinate system *xOy* is characterized by the coordinates *x*_3_ and *y*_2_. The wheels’ motion along the horizontal axis *Ox* is described by the coordinates *x*_5_ and *x*_6_. The position of the mass *m*_4_ can be uniquely defined by the parameters *x*_4_ and *y*_4_. In general, the robot’s locomotion can be modeled by two independent coordinates. Let us adopt *x*_2_ and *y*_2_ as the generalized coordinates. All the other parameters (*x*_3_, *x*_4_, *x*_5_, *x*_6_, *y*_4_) can be presented as the functions of the considered generalized coordinates:

$$x_{6}=x_{2};\;x_{3}=x_{5}=x_{2}-\sqrt{l_{BD}^{2}-{\left(y_{2\left(0\right)}+y_{2}\right)}^{2}};$$

$$x_{4}=x_{2}-0.5∙\sqrt{l_{BD}^{2}-{\left(y_{2\left(0\right)}+y_{2}\right)}^{2}};\;y_{4}=0.5∙\left(y_{2\left(0\right)}+y_{2}\right),$$ (1)

where *y*_2(0)_ is the initial vertical displacement of the hinge *B* relative to the horizontal axis tangential to the wheels. The radius of the wheels is considered negligibly small in comparison with the dimensions of the pantograph-type mechanism.

To develop the mathematical model describing the robot’s locomotion conditions, the Euler-Lagrange equations are used in the following form:

$$\frac{d}{dt}\left(\frac{\partial T}{\partial {\dot{x}}_{2}}\right)-\frac{\partial T}{\partial x_{2}}+\frac{\partial P}{\partial x_{2}}+\frac{\partial W}{\partial {\dot{x}}_{2}}=Q_{x_{2}};$$

$$\frac{d}{dt}\left(\frac{\partial T}{\partial {\dot{y}}_{2}}\right)-\frac{\partial T}{\partial y_{2}}+\frac{\partial P}{\partial y_{2}}+\frac{\partial W}{\partial {\dot{y}}_{2}}=Q_{y_{2}},$$ (2)

where *T* and *P* are the oscillatory system’s kinetic and potential energies, respectively; *W* is the energy dissipation function (Rayleigh dissipation function); ${\dot{x}}_{2}$ and ${\dot{y}}_{2}$ , $Q_{x_{2}}$ and $Q_{y_{2}}$ are the generalized velocities and the generalized forces, which correspond to the chosen generalized coordinates *x*_2_ and *y*_2_, respectively.

The total kinetic energy of the robot’s oscillatory system consists of the kinetic energies of six movable bodies: unbalanced mass (*T*_1_); robot’s body (*T*_2_); slider *A* (*T*_3_); mass *m*_4_ (*T*_4_); rear and front wheels (*T*_5_ and *T*_6_). The analytical expressions describing the corresponding kinetic energies are the following:

$$T_{1}=\frac{m_{1}∙{\left({\dot{x}}_{1}-r∙\omega ∙\sin\left(\omega ∙t\right)\right)}^{2}}{2}+\frac{m_{1}∙{\left({\dot{y}}_{1}+r∙\omega ∙\cos\left(\omega ∙t\right)\right)}^{2}}{2};$$

$$T_{2}=\frac{m_{2}∙{\left({\dot{x}}_{2}\right)}^{2}}{2}+\frac{m_{2}∙{\left({\dot{y}}_{2}\right)}^{2}}{2};\;T_{3}=\frac{m_{3}∙{\left({\dot{x}}_{3}\right)}^{2}}{2}+\frac{m_{3}∙{\left({\dot{y}}_{2}\right)}^{2}}{2};$$

$$T_{4}=\frac{m_{4}∙{\left({\dot{x}}_{4}\right)}^{2}}{2}+\frac{m_{4}∙{\left({\dot{y}}_{4}\right)}^{2}}{2};\;T_{5}=\frac{m_{5}∙{\left({\dot{x}}_{5}\right)}^{2}}{2};\;T_{6}=\frac{m_{6}∙{\left({\dot{x}}_{6}\right)}^{2}}{2};$$

$$T=T_{1}+T_{2}+T_{3}+T_{4}+T_{5}+T_{6},$$ (3)

where *r* is the eccentricity of the unbalanced mass ( $r=l_{KM}$ ); *ω* is the angular speed of the crank *KM* (motor’s shaft); *ω* is considered constant at the studied steady-state locomotion conditions.

The system’s potential energy is accumulated in the spring element *k* and can be determined as follows:

$$P=\frac{k∙{\left(x_{3}-x_{2}\right)}^{2}}{2}.$$ (4)

Let us assume that the energy dissipation is caused by a velocity-proportional frictional force, whose action upon the system can be described by the Rayleigh dissipation function:

$$W=\frac{c∙{\left({\dot{x}}_{3}-{\dot{x}}_{2}\right)}^{2}}{2}.$$ (5)

The generalized forces acting upon the system can be approximately modeled as the forces applied to the robot’s wheels. Neglecting the sliding and rolling friction conditions during the wheels motion, let us consider their unrestricted (free) motion in a rightward direction, and full blocking (unmovable state) when the wheels try to move leftward. Herewith, let us omit the robot’s motion condition when the wheels jump (bounce) over the supporting surface. Therefore, in the considered case, the expressions for determining $Q_{x_{2}}$ and $Q_{y_{2}}$ are as follows:

$$Q_{x_{2}}=\left\{\begin{array}{ll} 0,\;sign\left({\dot{x}}_{2}\right)\geq 0;\\ \frac{d}{dt}\left(\frac{\partial T}{\partial {\dot{x}}_{2}}\right)-\frac{\partial T}{\partial x_{2}}+\frac{\partial P}{\partial x_{2}}+\frac{\partial W}{\partial {\dot{x}}_{2}}-\sum_{i=1}^{i=6}m_{i}∙{\ddot{x}}_{2}, & sign\left({\dot{x}}_{2}\right)<0; \end{array}\right.\;Q_{y_{2}}=0,$$ (6)

where the signum function $sign\left({\dot{x}}_{2}\right)$ defines the horizontal speed sign of the robot’s body, i.e., its horizontal locomotion direction.

Substitution of Eq. 1, Eq. 3, Eq. 4, Eq. 5, and Eq. 6 into the differential Eq. 2 allows for developing the mathematical model, which describes the robot’s oscillatory system locomotion conditions. Since the obtained system of differential equations is huge (bulky), let us present it with substitutions as follows:

$$\begin{array}{ll} & \;m_{1}∙\left({\ddot{x}}_{2}-r∙\omega^{2}∙\cos\left(\omega ∙t\right)\right)+\left(m_{2}+m_{6}\right)∙{\ddot{x}}_{2}+\left(m_{3}+m_{5}\right)∙\left({\ddot{x}}_{2}+\xi \right)\\ & +m_{4}∙\left({\ddot{x}}_{2}+0.5∙\xi \right)= \end{array}$$

$$\;\left\{\begin{array}{ll} 0,\;sign\left({\dot{x}}_{2}\right)\geq 0;\\ -m_{1}∙r∙\omega^{2}∙\cos\left(\omega ∙t\right)+\left(m_{3}+0.5∙m_{4}+m_{5}\right)∙\xi , & sign\left({\dot{x}}_{2}\right)<0; \end{array}\right.$$

$$\begin{array}{ll} & \;m_{1}∙\left({\ddot{y}}_{2}-r∙\omega^{2}∙\sin\left(\omega ∙t\right)\right)+m_{2}∙{\ddot{y}}_{2}+m_{3}∙\left({\ddot{y}}_{2}+\zeta \right)+0.25∙\\ & \;m_{4}∙\left({\ddot{y}}_{2}+\zeta +\chi \right)+m_{5}∙\zeta =0. \end{array}$$ (7)

where:

$$\xi =\frac{\left(y_{2\left(0\right)}+y_{2}\right)∙{\ddot{y}}_{2}+{\dot{y}}_{2}^{2}}{\sqrt{l_{BD}^{2}-{\left(y_{2\left(0\right)}+y_{2}\right)}^{2}}}+\frac{{\left(y_{2\left(0\right)}+y_{2}\right)}^{2}∙{\dot{y}}_{2}^{2}}{\sqrt{{\left(l_{BD}^{2}-{\left(y_{2\left(0\right)}+y_{2}\right)}^{2}\right)}^{3}}};$$ (8)

$$\begin{array}{ll} \zeta & =\left(y_{2\left(0\right)}+y_{2}\right)∙\left(l_{BD}^{2}∙{\dot{y}}_{2}^{2}+\left(l_{BD}^{2}-{\left(y_{2\left(0\right)}+y_{2}\right)}^{2}\right)∙\left(\sqrt{l_{BD}^{2}-{\left(y_{2\left(0\right)}+y_{2}\right)}^{2}}\right.\right.\\ & \;\left.\left.∙{\ddot{x}}_{2}+\left(y_{2\left(0\right)}+y_{2}\right)∙{\ddot{y}}_{2}\right)\right)/{\left(l_{BD}^{2}-{\left(y_{2\left(0\right)}+y_{2}\right)}^{2}\right)}^{2}; \end{array}$$ (9)

$$\chi =\frac{{\ddot{x}}_{2}∙\sqrt{l_{BD}^{2}-{\left(y_{2\left(0\right)}+y_{2}\right)}^{2}}∙\left(l_{BD}^{2}-{\left(y_{2\left(0\right)}+y_{2}\right)}^{2}\right)∙\left(y_{2\left(0\right)}+y_{2}\right)}{{\left(l_{BD}^{2}-{\left(y_{2\left(0\right)}+y_{2}\right)}^{2}\right)}^{2}}.$$ (10)

### 2.3 Experimental technique and equipment

The experiments are carried out at the Vibroengineering Laboratory of Lviv Polytechnic National University. The robot’s experimental prototype 1 is implemented in practice and equipped with the inertial vibration exciter SL-VBM-3660C-12 (Shanglin Motor Co., Ltd). The exciter 3 is installed on the main plate of the robot’s body, where the accelerometer BWT901CL (WitMotion Shenzhen Co., Ltd) is fixed. The forced frequency of the exciter (the angular speed of the DC motor’s shaft) is regulated by the voltage supplied to the motor. For this purpose, the controllable power adaptor QiYe-32430 ZY-009 (Zuczug–Shenzhen Aotus Electronics Co., Ltd) is used. The latter converts the alternating voltage of 220 V (50 Hz) available in the power supply socket 5 to the constant (direct) voltage of adjustable value within 3 … 24 V. The experimental data registered by the accelerometer 2 is sent to the laptop 6 using the USB-C cable. The WitMotion software installed on the laptop 6 is used for processing the accelerometer data and writing them into text files suitable for further analysis in Microsoft Excel, PTC MathCad, Maplesoft Maple, Wolfram Mathematica, or other software.

The experiments are conducted at eight voltage values: 3.47, 4, 5, 6, 7, 8, 9, 10 V. At a voltage lower than 3.47 V, the robot does not move. Increasing the voltage value over 10 V causes the robot to jump (bounce) over the supporting surface. The dependences of the robot body’s vertical and horizontal accelerations and the shaft’s angular speed (forced frequency) on the voltage supplied to the DC motor are experimentally tested using the WitMotion sensor. The average robot’s speed is calculated as the ratio between the distance passed by the robot during the time interval of 10 s measured by the stopwatch. In addition, the experimental data obtained in the WitMotion software is processed in the MathCad software. The numerical integration using the built-in Runge-Kutta methods is carried out to determine time dependencies of the robot body’s vertical and horizontal speeds and displacements. The comparative analysis of the results of the accelerometer data numerical integration and the results of measuring the robot’s average translational speed is conducted.

## 3 Results and discussion

### 3.1 Numerical modeling of the system vibrations in the Mathematica software

**FIGURE 3:**
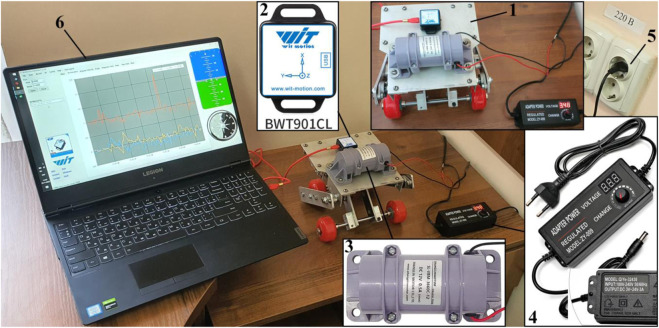
Laboratory equipment used for conducting experimental investigations: **(1)**—robot’s experimental prototype; **(2)**—accelerometer; **(3)**—vibration exciter; **(4)**—voltage regulator; **(5)**—power supply socket; **(6)**—laptop.

As it is shown below, based on the experimental tests, the considered voltage values (3.47, 4, 5, 6, 7, 8, 9, 10 V) provide the following angular frequencies of the DC motor’s shaft: 500, 580, 680, 860, 990, 1,160, 1,350, 1,500 rpm, respectively. While performing numerical modeling, let us adopt eight cyclic frequencies ω : 52.4 s^−1^ (500 rpm), 60.7 s^−1^ (580 rpm), 71.2 s^−1^ (680 rpm), 90.1 s^−1^ (860 rpm), 103.7 s^−1^ (990 rpm), 121.5 s^−1^ (1,160 rpm), 141.4 s^−1^ (1,350 rpm), and 157.1 s^−1^ (1,500 rpm). All the geometrical, inertial, stiffness, and damping parameters are obtained based on the robot’s 3D-design and experimental prototype (Figure 1; Figure 3): $m_{1}=0.099kg$ , $m_{2}=0.841kg$ , $m_{3}=0.262kg$ , $m_{4}=0.584kg$ , $m_{5}=m_{6}=0.223kg$ , $l_{AC}=l_{BC}=l_{CD}=l_{CE}=0.07m$ , $l_{KM}=r=0.003m$ . The initial distance between the hinges *A* and *B* (*D* and *E*) at the static equilibrium conditions is the following: $l_{AB0}=l_{DE0}=0.108m$ . The initial distance between the hinges *A* and *D* (*B* and *E*) at the static equilibrium conditions is considered as: $y_{2\left(0\right)}=0.089m$ . The total stiffness coefficient of the spring elements of the robot’s suspension is the following: $k=2.3∙10^{3}N/m$ , and the reduced damping (viscous friction) coefficient is approximately equal to c=30N∙s/m .

**FIGURE 4:**
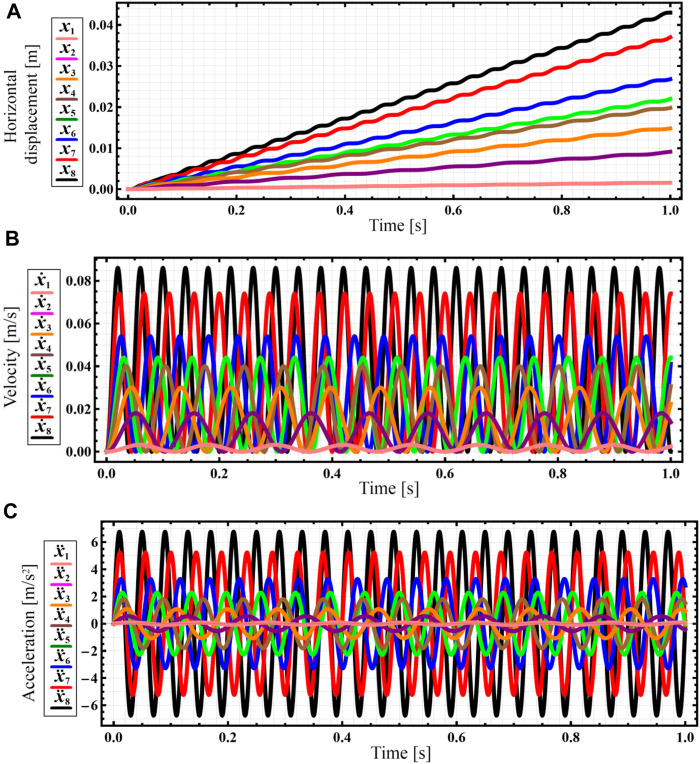
Results of numerical modeling of the robot’s steady-state locomotion conditions carried out in the Mathematica software (indexes 1 … 8 define the kinematic parameters at the corresponding forced frequencies: 1–52.4 s^−1^; 2–60.7 s^−1^; 3–71.2 s^−1^; 4–90.1 s^−1^; 5–103.7 s^−1^; 6–121.5 s^−1^; 7–141.4 s^−1^; 8–157.1 s^−1^). **(A)** horizontal displacement; **(B)** horizontal velocity; **(C)** horizontal acceleration.

The numerical integration of the differential Eq. 7 is carried out in the Mathematica software with the help of the built-in Runge-Kutta methods. The corresponding results of numerical modeling of the robot’s steady-state locomotion conditions are shown in Figure 4 in the form of time response curves of the distance traveled by the robot, its speed, and acceleration. Eight curves [*x*_1_(*t*)…*x*_8_(*t*)] correspond to the forced frequencies considered above. The largest horizontal displacements, speeds, and accelerations are observed under the conditions of the largest forced frequency of 157.1 s^−1^ or 1,500 rpm (see black curves in Figure 4). In this case, the robot travels a distance of about 0.043 m during the time interval of 1 s. Thus, its average locomotion speed is approximately 0.043 m/s. The amplitude values of the corresponding speed and acceleration exceed 0.082 m/s and 6.3 m/s^2^. The smallest displacements, speeds, and accelerations are obtained at the smallest forced frequency of 52.4 s^-1^ or 500 rpm (see pink curves in Figure 4). At these conditions, the robot passes a distance of approximately 0.0012 m during the time interval of 1 s. Thus, its average horizontal speed is about 0.0012 m/s. The amplitude values of the corresponding speed and acceleration reach 0.0024 m/s and 0.1 m/s^2^.

Further computer simulation (virtual experiment) will be performed at a forced frequency of 141.4 s^-1^ (1,350 rpm), which allows the robot to reach the average locomotion speed of approximately 0.037 m/s (see red curve in Figure 4A), whereas the amplitude values of the horizontal speed and acceleration are about 0.074 m/s and 5 m/s^2^, respectively (see red curves in Figures 4B,C).

### 3.2 Computer simulation of the robot motion in the SolidWorks software

**FIGURE 5:**
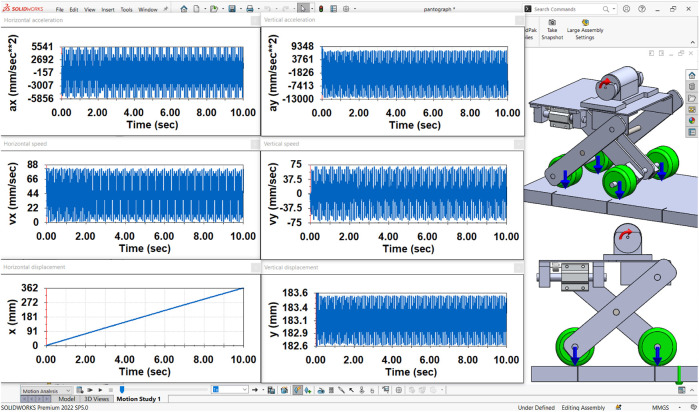
Simulation results obtained in the SolidWorks Motion software.

In order to check the correctness of the numerical modeling results, the computer simulation of the robot’s locomotion conditions is performed in the SolidWorks Motion software using the simplified robot’s 3D-model (virtual prototype) and the built-in variable-step integration method (Gear’s method). The input parameters (inertial, stiffness, and damping characteristics, geometrical parameters, etc.) correspond to the ones used for numerical modeling. The simulation results presenting the robot’s basic kinematic characteristics at the forced frequency of 22.5 Hz (1,350 rpm) are shown in Figure 5. The horizontal acceleration of the robot’s body varies in the range of about −5,500 … 5,500 mm/s^2^, while the magnitudes of the maximal and minimal vertical accelerations do not exceed 9,000 mm/s^2^ and –13000 mm/s^2^, respectively. Considering the horizontal speed, its lowest values do not decrease to less than zero, while the peak values are about 80 mm/s. The robot’s body’s vertical speed varies in the range of about −70 … 70 mm/s. The amplitude of vertical oscillations is approximately 0.5 mm (peak-to-peak value equals 1 mm). During the time period of 10 s, the robot’s body traveled a distance of about 362 mm in a horizontal direction. Therefore, its average horizontal speed is equal to 0.0362 m/s. The obtained simulation results (Figure 5) satisfactorily agree with the corresponding numerical modeling results presented above (see Figure 4). Some differences in the maximal and minimal values of the robot’s displacements, speeds, and accelerations do not exceed 10% and can be explained by the errors of the numerical methods used for solving the differential equation and performing the computer simulation.

### 3.3 Experimental testing of the robot’s kinematic parameters at different forced frequencies

**FIGURE 6:**
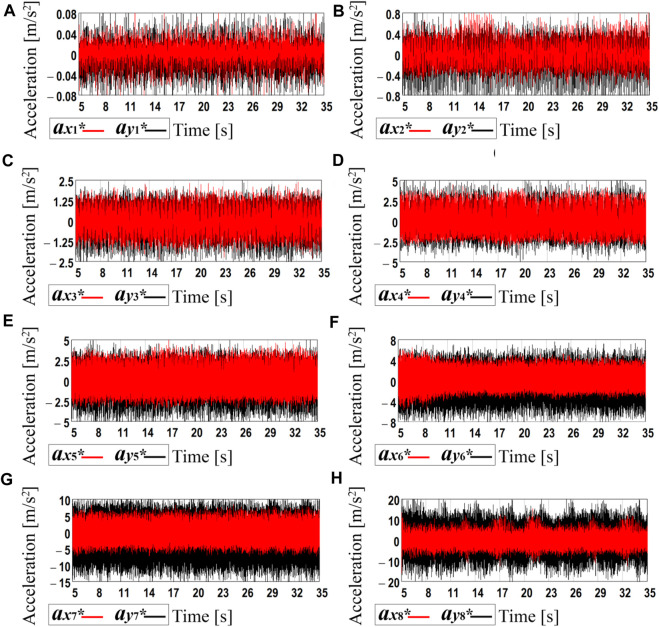
Experimental data registered by the accelerometer during the robot motion at different forced frequencies: **(A)** 52.4 s^−1^ (500 rpm); **(B)** 60.7 s^−1^ (580 rpm); **(C)** 71.2 s^−1^ (680 rpm); **(D)** 90.1 s ^−1^ (860 rpm); **(E)** 103.7 s^−1^ (990 rpm); **(F)** 121.5 s^−1^ (1,160 rpm); **(G)** 141.4 s^−1^ (1,350 rpm); **(H)** 157.1 s^−1^ (1,500 rpm).

**FIGURE 7:**
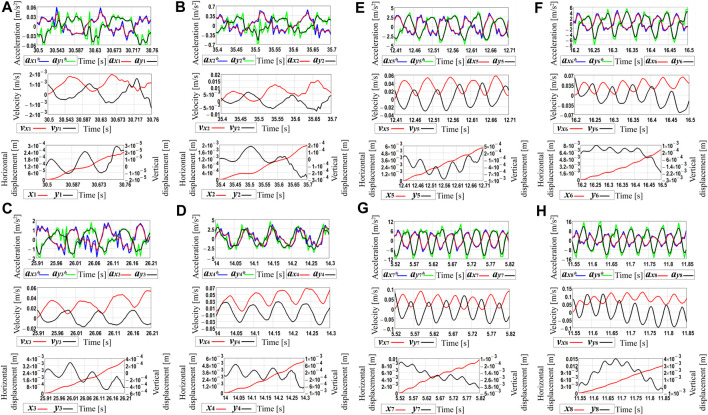
Robot’s kinematic characteristics obtained by processing the experimental data in the MathCad software at different values of the forced frequencies: **(A)** 52.4 s^−1^ (500 rpm); **(B)** 60.7 s ^−1^ (580 rpm); **(C)** 71.2 s^−1^ (680 rpm); **(D)** 90.1 s^−1^ (860 rpm); **(E)** 103.7 s^−1^ (990 rpm); **(F)** 121.5 s ^−1^ (1,160 rpm); **(G)** 141.4 s^−1^ (1,350 rpm); **(H)** 157.1 s^−1^ (1,500 rpm).

Further research on the subject of the paper is focused on experimental investigations of the robot’s locomotion conditions. The results of the eight laboratory tests carried out at the supplied voltage values of 3.47, 4, 5, 6, 7, 8, 9, 10 V are presented in Figure 6. The WitMotion sensors and software showed that the amplitude values of the robot’s body horizontal acceleration vary from approximately 0.06 m/s^2^ at the voltage of 3.47 V (see curve *a*_*x*1_* in Figure 6A) to 7 m/s^2^ at 10 V (see curve *a*_*x*8_* in Figure 6H). Considering the vertical acceleration, its maximal values are about 0.07 m/s^2^ at 3.47 V (see curve *a*_*y*1_* in Figure 6A) and 14 m/s^2^ at 10 V (see curve *a*_*y*8_* in Figure 6H). In general, the increase in the supplied voltage causes the augmentation of the forced frequency and centrifugal acceleration of the unbalanced rotor. This provides an increase in the robot body’s horizontal and vertical accelerations.

In order to determine the time dependencies of the robot body’s horizontal and vertical speeds and displacements, the processing of the experimental data was carried out in the MathCad software. Due to the limited calculational possibilities of the available hardware, the time interval of 0.3 s was chosen for each plot of Figure 6. The corresponding acceleration data were numerically integrated using the Runge-Kutta methods. In such a way, the time dependencies of the robot’s body horizontal and vertical speeds and displacements were obtained (see Figure 7). The blue and green curves correspond to the real experimental data (see curves *a*_*x*1_*…*a*_*x*8_*, *a*_*y*1_*…*a*_*y*8_*), whereas the corresponding red and black curves are obtained by numerical interpolation and integration of the experimental data. The amplitude values of the horizontal speed vary from approximately 2 mm/s at the voltage of 3.47 V (see curve *v*_*x*1_ in Figure 6A) to 125 mm/s at 10 V (see curve *v*_*x*8_ in Figure 6H). Considering the vertical speed, its maximal values are about 0.1 mm/s at 3.47 V (see curve *v*_*y*1_ in Figure 6A) and 110 mm/s at 10 V (see curve *v*_*y*8_ in Figure 6H). During the time interval of 0.3 s, the robot’s body passed the horizontal distance of 0.24 mm at the supplied voltage of 3.47 V (see curve *x*_1_ in Figure 6A) and the distance of 1.2 mm at 10 V (see curve *x*_8_ in Figure 6H). Therefore, it can be concluded that the robot’s average locomotion speed varies from 0.8 mm/s (at 3.47 V) to 4 mm/s (at 10 V). Due to the small time interval used for analyzing the robot locomotion conditions, the obtained numerical interpolation and integration results to some extent are different from the results of numerical modeling and computer simulation. Therefore, further investigations on the average robot’s speed are carried out. The latter is calculated as the ratio between the distance passed by the robot during the time interval of 10 s measured by the stopwatch.

### 3.4 Discussion

**FIGURE 8:**
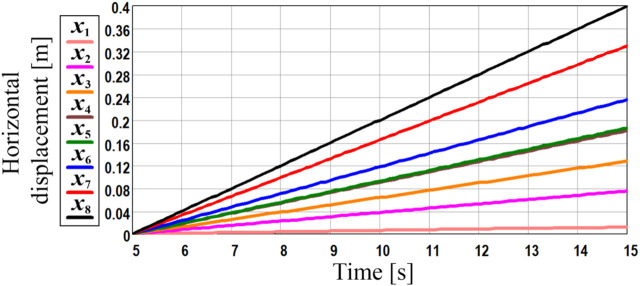
General character of the robot locomotion during the time interval of 10 s.

The last stage of the experimental investigations is dedicated to analyzing the horizontal distance traveled by the robot within 10 s at different supplied voltages with the help of the video recording of the robot locomotion along the horizontal surface. This study allowed for defining the average locomotion speeds of the robot under different excitation conditions. Figure 8 presents the general character of the robot locomotion within the time range of 5 … 15 s after the start, particularly, the horizontal distance passed by the robot during the time interval of 10 s. The obtained results showed that the robot’s horizontal displacement almost linearly depends on the elapsed time. This allows for concluding about the approximately constant locomotion speed of the robot. The largest displacement of about 0.4 m is observed at the supplied voltage of 10 V, whereas the smallest distance traveled by the robot is approximately 0.01 m at the supplied voltage of 3.47 V.

**FIGURE 9:**
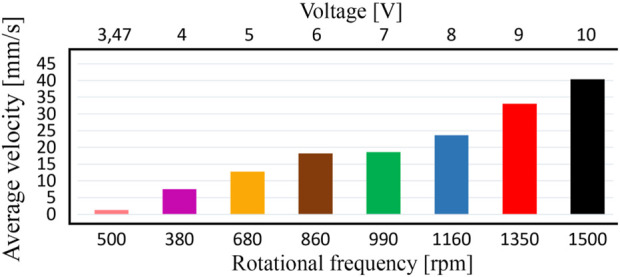
Dependence of the robot’s average locomotion speed on the supplied voltage and forced frequency.

The obtained experimental results satisfactorily agree with the ones obtained during the numerical modeling and computer simulation. Considering the previously discussed case when the forced frequency is equal to 22.5 Hz (the unbalanced shaft rotates at 1,350 rpm), the numerical modeling and computer simulation showed that the robot’s average horizontal speed is about 0.036 … 0.037 m/s (see Figure 4; Figure 5). The experimental data obtained at the supplied voltage of 9 V corresponds to the forced frequency of 1,350 rpm (see Figure 7G; red curve *x*_7_ at Figure 8; Figure 9). During the time period of 10 s, the robot’s body passed the horizontal distance of about 0.33 m. Therefore, its average locomotion speed is approximately 0.033 m/s. The difference between the results of experimental investigations, numerical modeling, and computer simulation is about 10% and can be explained by the energy losses due to friction in the mechanical system of the robot’s suspension. In general, the smallest horizontal speed of about 1 mm/s is observed at the supplied voltage of 3.47 V when the forced frequency is equal to 500 rpm. The largest locomotion speed is approximately 40 mm/s at the supplied voltage of 10 V and forced frequency of 1,500 rpm.

The performed investigations substantiate the possibilities of implementing the enhanced pantograph-type suspension of the wheeled vibration-driven robot and present the results of numerical modeling, computer simulation, and experimental investigations of the robot’s kinematic characteristics under different excitation conditions. The proposed robot’s suspension, mathematical and simulation models, as well as the obtained results can be used by designers and researchers of similar vibration-driven locomotion systems while implementing their experimental and industrial prototypes for various purposes, particularly, for monitoring and cleaning the pipelines. In distinction to the existent papers devoted to the wheeled vibration-driven robots, in particular, (Korendiy et al., 2022a; Korendiy et al., 2022b; Korendiy et al., 2022c; Korendiy et al., 2022d; Rusu and Tatar, 2022; Tagliavini et al., 2022; Loukanov, 2015; Loukanov and Stoyanov, 2015; Loukanov et al., 2016a; Loukanov et al., 2016b; Loukanov, 2016; Korendiy et al., 2022f; Korendiy et al., 2022g; Korendiy et al., 2023), the proposed improved design of the pantograph-type locomotion system allows for efficient using both horizontal and vertical components of the centrifugal forces generated due to the unbalanced mass rotation and actuating the robot’s body. The vast majority of the existent vibration-driven robots with centrifugal (inertial) exciters effectively utilize only the horizontal components of the disturbing forces, while the proposed system allows for increasing the operational efficiency of the exciter. This influences the robot’s power consumption and average translational velocity. In addition, it is necessary to mention that the general locomotion character and operating conditions of the considered robot correspond to the ones previously modeled, simulated, and experimentally tested in (Korendiy et al., 2022a; Korendiy et al., 2022b; Korendiy et al., 2022c; Korendiy et al., 2022d; Rusu and Tatar, 2022; Tagliavini et al., 2022; Loukanov, 2015; Loukanov and Stoyanov, 2015; Loukanov et al., 2016a; Loukanov et al., 2016b; Loukanov, 2016; Korendiy et al., 2022f; Korendiy et al., 2022g; Korendiy et al., 2023). In the considered literature, the average speed of similar robotic systems does not exceed 20 … 36 mm/s, while the proposed wheeled vibration-driven robot with the enhanced pantograph-type suspension can reach an average speed of over 40 mm/s. Further increase in the supplied voltage over 10 V and the forced frequency over 1,500 rpm causes the occurrence of the jumping (bouncing) locomotion conditions when the robot’s wheels start detaching (rising above) the supporting surface and the robot’s average horizontal speed significantly reduces. This phenomenon will be comprehensively studied while performing further investigations.

## 4 Conclusions and further investigations

The present research is a logical extension of the authors’ previous investigations on the dynamic behavior of the wheeled vibration-driven robots equipped with centrifugal vibration exciters. The central theme of this paper is focused on the enhanced pantograph-type suspension which is followed by deriving a new mathematical model describing the robot’s locomotion conditions. Specific attention is paid to numerical modeling of the robot dynamics at different excitation conditions, particularly, forced frequencies, using the Mathematica software. The verification of the theoretical investigations is performed by computer simulation of the robot locomotion in the SolidWorks software. The last stage of the research is dedicated to experimental investigations of the robot’s full-scale prototype at the Vibroengineering Laboratory of Lviv Polytechnic National University.

The investigations are carried out at different supplied voltages of 3.47, 4, 5, 6, 7, 8, 9, 10 V defining the corresponding forced frequencies (angular frequencies of the unbalanced rotor) of about 500, 580, 680, 860, 990, 1,160, 1,350, 1,500 rpm. The obtained results are presented in the form of time response curves of the robot body’s basic kinematic characteristics: horizontal and vertical accelerations, speeds, and displacements. The numerical modeling, computer simulation, and experimental investigations showed almost similar results: the smallest horizontal speed of about 1 mm/s is observed at the supplied voltage of 3.47 V when the forced frequency is equal to 500 rpm; the largest locomotion speed is approximately 40 mm/s at the supplied voltage of 10 V and forced frequency of 1,500 rpm. The increase in the supplied voltage causes the augmentation of the forced frequency and centrifugal acceleration of the unbalanced rotor. This provides the increase in the robot body’s horizontal and vertical accelerations, as well as the increase in its average locomotion speed. Further increase in the supplied voltage over 10 V and the forced frequency over 1,500 rpm causes the occurrence of the jumping (bouncing) locomotion conditions when the robot’s wheels start detaching (rising above) the supporting surface and the robot’s average horizontal speed significantly reduces.

The proposed robot’s suspension, mathematical and simulation models, as well as the obtained results can be used by designers and researchers of similar vibration-driven locomotion systems while implementing their experimental and industrial prototypes for various purposes, particularly, for inspecting and cleaning the pipelines. Further investigation on the subject of the paper should be focused on analyzing the relations between the power consumption, average translational speed, and working efficiency of the considerer robot under various operating conditions. In addition, it is necessary to define the advantages and drawbacks of the proposed design of the wheeled robot in comparison with other types of vibration-driven locomotion systems and to analyze the possibilities and directions of further improvement of the considered pantograph-type suspension. As well, further investigations should deal with analyzing the conditions of occurrence of the jumping (bouncing) locomotion regimes when the robot’s wheels start detaching (rising above) the supporting surface.

## Data Availability

The original contributions presented in the study are included in the article/, further inquiries can be directed to the corresponding author.
